# Deciphering the Molecular Basis of Melatonin Protective Effects on Breast Cells Treated with Doxorubicin: TWIST1 a Transcription Factor Involved in EMT and Metastasis, a Novel Target of Melatonin

**DOI:** 10.3390/cancers11071011

**Published:** 2019-07-19

**Authors:** Javier Menéndez-Menéndez, Francisco Hermida-Prado, Rocío Granda-Díaz, Alicia González, Juana María García-Pedrero, Nagore Del-Río-Ibisate, Alicia González-González, Samuel Cos, Carolina Alonso-González, Carlos Martínez-Campa

**Affiliations:** 1 Department of Physiology and Pharmacology, School of Medicine, University of Cantabria and Instituto de Investigación Valdecilla (IDIVAL), 39011 Santander, Spain; 2 Department of Otolaryngology, Hospital Universitario Central de Asturias and Instituto de Investigación Sanitaria del Principado de Asturias, 33011 Oviedo, Spain; 3 Instituto Universitario de Oncología del Principado de Asturias, University of Oviedo, 33011 Oviedo, Spain

**Keywords:** melatonin, breast cancer, doxorubicin, chemotherapy, MCF-7 cells, TWIST1, p70S6K, miR-10b

## Abstract

Melatonin mitigates cancer initiation, progression and metastasis through inhibition of both the synthesis of estrogens and the transcriptional activity of the estradiol-ER (Estrogen receptor) complex in the estrogen-dependent breast cancer cell line MCF-7. Moreover, melatonin improves the sensitivity of MCF-7 to chemotherapeutic agents and protects against their side effects. It has been described that melatonin potentiates the anti-proliferative effects of doxorubicin; however, the molecular changes involving gene expression and the activation/inhibition of intracellular signaling pathways remain largely unknown. Here we found that melatonin enhanced the anti-proliferative effect of doxorubicin in MCF-7 but not in MDA-MB-231 cells. Strikingly, doxorubicin treatment induced cell migration and invasion, and melatonin effectively counteracted these effects in MCF-7 but not in estrogen-independent MDA-MB-231 cells. Importantly, we describe for the first time the ability of melatonin to downregulate TWIST1 (Twist-related protein 1) in estrogen-dependent but not in estrogen-independent breast cancer cells. Combined with doxorubicin, melatonin inhibited the activation of p70S6K and modulated the expression of breast cancer, angiogenesis and clock genes. Moreover, melatonin regulates the levels of TWIST1-related microRNAs, such as miR-10a, miR-10b and miR-34a. Since TWIST1 plays a pivotal role in the epithelial to mesenchymal transition, acquisition of metastatic phenotype and angiogenesis, our results suggest that inhibition of TWIST1 by melatonin might be a crucial mechanism of overcoming resistance and improving the oncostatic potential of doxorubicin in estrogen-dependent breast cancer cells.

## 1. Introduction

Melatonin is an indolamine mainly secreted by the pineal gland during the night. It is a multitasking molecule acting through various mechanisms (e.g., G-protein coupled membrane receptors, interactions with orphan receptors or cytosolic molecules such as calmodulin), thereby playing multiple different roles as an antioxidant, immunomodulatory, endocrine regulatory molecule, circadian rhythm synchronizer or anticancer agent controlling cell physiology [1,2,3]. Melatonin prevents cancer at the initiation, tumor progression and metastatic dissemination [4,5]. In particular, the oncostatic actions of melatonin have been largely described in hormone-dependent tumors, either when administered alone or in combination with chemotherapeutic agents [6,7,8].

In breast cancer, the pineal hormone controls the transcriptional activity of the estradiol-estrogen receptor complex, therefore behaving as a selective estrogen receptor modulator (SERM), and also diminishes the production of estrogens through the regulation of the enzymes involved in estrogen activation/inactivation, thus behaving as a selective estrogen enzyme modulator (SEEM) [9,10,11]. 

Melatonin has been tested in combination with different molecules and, as such, beneficial effects have been demonstrated in preclinical studies [12], in vivo animal models [13,14] and in vitro cellular models [15]. 

Melatonin sensitizes the estrogen-dependent breast cancer cell line MCF-7 to doxorubicin by increasing its intracellular concentration, probably through inhibition of the P-glycoprotein [16]. Conversely, disruption of nocturnal melatonin driven by light-at-night in a model of MCF-7 cancer xenografts grown in nude rats induces doxorubicin resistance that involves inhibition of phospho-AKT activation. Importantly, melatonin acts dually as a tumor inhibitor and a circadian regulatory molecule, thereby contributing to re-establish the sensitivity of breast tumors to doxorubicin, probably by suppression of the Warburg effect (related to phospho-activation of AKT) and by inhibition of various transcription factors such as STAT3 and NF-kB and proliferation and survival pathways including the EGF and ERK families [17]. In both MCF-7 cells and rats bearing ER-responsive mammary tumors, melatonin cooperates with doxorubicin resulting in lighter tumor weights, increased apoptosis, higher expression of E-cadherin and higher survival rate, therefore indicating that melatonin potentiates tumor sensitivity to doxorubicin [18,19].

However, the molecular basis of the protective role of melatonin and the impact on doxorubicin response of breast cancer cells still largely remain to be elucidated. This prompted us to perform a thorough analysis to decipher the functional role of melatonin on doxorubicin-treated breast cancer cells and to identify the molecular mechanisms and signaling pathways involved in the regulation by melatonin. We found that melatonin enhanced the anti-proliferative effect of doxorubicin and counteracted the stimulatory effect of the anthracycline on cell migration and invasion in MCF-7 cells. The pineal hormone inhibited activation of the Akt and p70S6 kinases and modulated multiple changes in the expression of cancer, angiogenesis and clock genes. Importantly, we describe for the first time the ability of melatonin to counteract the stimulatory effect of doxorubicin on TWIST1 (Twist-related protein 1) expression and protein levels in estrogen-dependent but not in estrogen-independent breast cancer cells. TWIST1 is involved in epithelial to mesenchymal transition and cancer metastasis and its up-regulation correlates to poor survival [20,21,22]. 

Finally, we found that melatonin produced a significant regulation of several TWIST1-related miRNAs which expression was altered by doxorubicin. Among them, melatonin was able to revert the stimulatory effect of doxorubicin on the expression of miR-10b, known to be induced by TWIST1, thus contributing to the acquisition of the metastatic phenotype and stimulation of angiogenesis [23,24]. 

Our results revealed that melatonin modulated multiple changes induced by doxorubicin, and we found, for the first time, that melatonin counteracted the stimulatory effect of doxorubicin on the expression of TWIST1 in breast cancer cells.

## 2. Results

### 2.1. Effects of Melatonin and Doxorubicin on the Proliferation of MCF-7 and MDA-MB-231 Cells

We first investigated the ability of melatonin to modulate the anti-proliferative effects of doxorubicin, by using two different breast cancer cell lines: the hormone-dependent MCF-7 and the hormone-independent MDA-MB-231.

**Figure 1:**
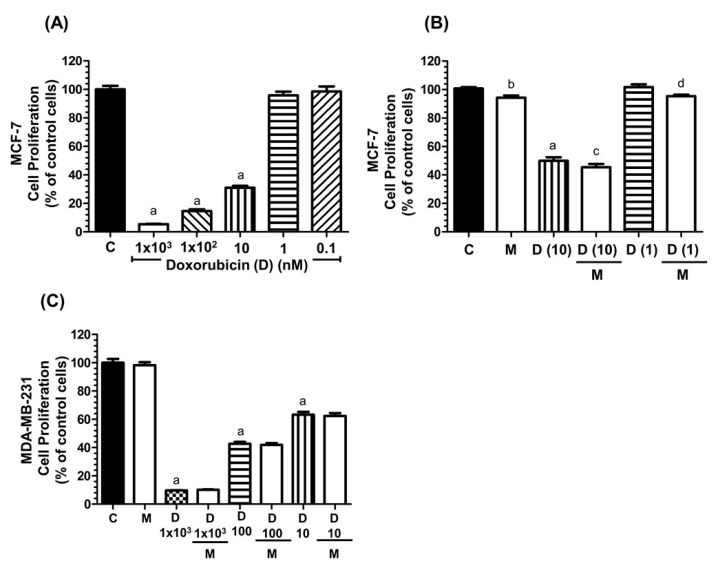
Effects of doxorubicin (D) and melatonin (M) on the proliferation of breast cancer cells. Cell proliferation was measured by the MTT [3(4,5-dimethylthiazol-2-yl)-2,5-diphenyl tetrazolium bromide] method after 3 days of incubation with the different treatments specified below. (**A**) MCF-7 cells were seeded into 96-well plates in Dulbecco’s Modified Eagle’s Medium (DMEM) supplemented with 10% FBS and treated with vehicle (control) or doxorubicin (ranging from 1 µM to 0.1 nM). (**B**) MCF-7 cells were seeded into 96-multiwell plates in DMEM supplemented with 10% FBS and treated with doxorubicin (10 nM or 1 nM) alone or in combination with melatonin (1 nM) and incubated at 37 °C for 3 days. Cell proliferation was measured by the MTT method. (**C**) MDA-MB-231 cells were seeded into 96-multiwell plates in DMEM supplemented with 10% FBS and treated with doxorubicin (ranging from 1 mM to 10 nM) alone or in combination with melatonin (1 nM). a, *p* < 0.001 vs. C; b, *p* < 0.05 vs. C; c, *p* < 0.01 vs. D (10 nM); d, *p* < 0.01 vs. D (1 nM).

As expected, doxorubicin inhibited the proliferation of MCF-7 cells in a dose dependent manner (Figure 1A). We next tested the effect of the addition of physiological doses of melatonin (1 nM) to cell cultures treated with doxorubicin ranging from 10 to 1 nM. After 3 days of treatment, the proliferation of MCF-7 cells decreased at doxorubicin concentrations of 5 and 10 nM. Lower concentrations of doxorubicin had no effect on cell proliferation. Co-treatment with a physiological dose of melatonin (1 nM) led to a significant further decrease in cell proliferation compared to cells treated with doxorubicin alone (Figure 1B). However, in the triple-negative breast cancer cell line MDA-MB-231, co-treatment with melatonin (1 nM) did not significantly alter the inhibitory effect of doxorucibin at any of the (even high) doses tested (Figure 1C).

### 2.2. Effects of Melatonin and Doxorubicin on Cell Migration and Invasion in MDA-MB-231 and MCF-7 Cells 

**Figure 2:**
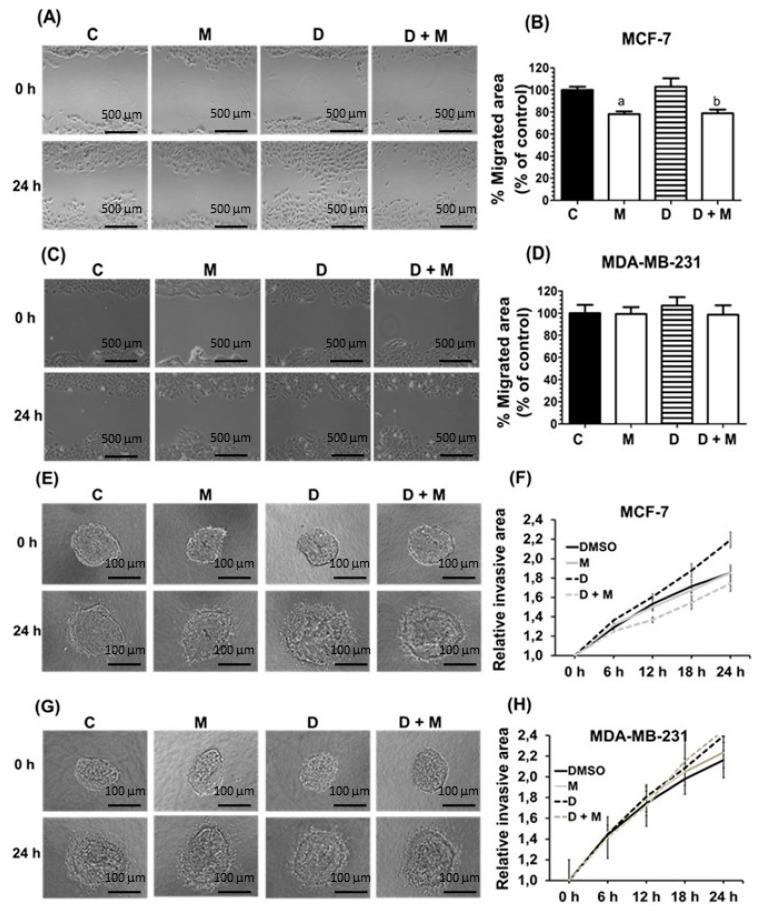
Effect of melatonin and doxorubicin on migration and on the invasive potential of MCF-7 and MDA-MB-231 cells. **A**, **C** Effects of melatonin (1 nM) and doxorubicin (1 µM) on MCF-7 (**A**) or MDA-MB-231 (**C**) cell migration analyzed through the wound healing assay. Quantification of MCF-7 (**B**) or MDA-MB-231 (**D**) cell migration was expressed as mean ± SEM. Representative microphotographs of initial and after 24 h are shown. (**E**,**G**) Effects of melatonin (1 nM) and doxorubicin (1 µM) on MCF-7 (**E**) or MDA-MB-231 (**G**) invasive potential. Representative images from the 3D invasion assays of cell spheroids embedded into a collagen matrix at initial (*t* = 0 h) and final time (*t* = 24 h) for the different treatments are shown. (**F**,**H**) Graphs represent the quantification of the invasive area of MDA-MB-231 (**F**) or MCF-7 (**H**) cells at the indicated times. Data was expressed as mean ± SEM. **A**, **C**: Scale bar: 500 µm; **E**, **G**: Scale bar: 100 µm.

We next investigated the effects of doxorubicin and melatonin on the migratory capacity of MCF-7 and MDA-MB-231 cells by using wound-healing assays. As shown in Figure 2A,B, doxorubicin treatment did not alter cell migration in MCF-7 cells, whereas melatonin significantly decreased cell migration either alone or in combination with doxorubicin. In marked contrast, neither doxorubicin nor melatonin had any effect on the migratory capacity of MDA-MB-231 cells (Figure 2C,D). When the invasive potential was tested, we found that doxorubicin enhanced the invasive potential of MCF-7 cells, whereas addition of melatonin counteracted this stimulatory effect (Figure 2G,H). In contrast, the invasive potential of MDA-MB-231 was not altered by doxorubicin, and melatonin treatment did not have any significant effect (Figure 2E,F).

### 2.3. Effects of Doxorubicin and Melatonin on the Expression of Cancer-Related Genes

We used the human breast cancer RT² Profiler PCR Array to assess the expression changes in MCF-7 cells upon treatment with doxorubicin (1 µM) either alone or combined with a physiological dose of melatonin (1 nM). The RT² Profiler PCR Array allows the simultaneous analysis of 84 genes involved in various different key processes for breast cancer biology, such as angiogenesis, cell adhesion, proteases, breast cancer classification markers, signal transduction, cell cycle, transcription factors, apoptosis, DNA damage and repair. As shown in Table 1, doxorubicin alone upregulated the expression of 27 genes and downregulated 17 genes.

**Figure 3:**
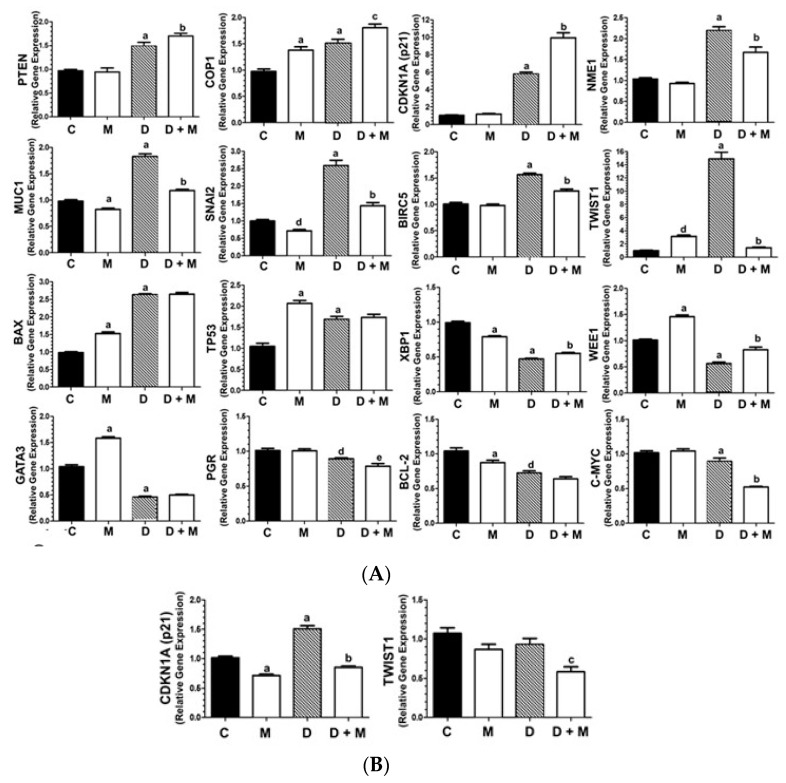
Gene expression profile of breast cancer related genes. (**A**) Validation of gene expression changes of selected 16 genes (>1.5 fold-change) by specific RT-PCR in MCF-7 cells treated with doxorubicin (1 µM) and/or melatonin (1 nM). All data are expressed as fold changes relative to vehicle-treated (control) cells (mean ± SEM) from three independent experiments. (**B**) RT-PCR analysis of *TWIST1* and *CDKN1A* genes in MDA-MB-231 cells treated with doxorubicin (1 µM) and/or melatonin (1 nM) for 6 h. Data are expressed as fold changes relative to control cells (mean ± SEM) from three independent experiments. a, *p* < 0.001 vs. C; b, *p* < 0.001 vs. D; c, *p* < 0.01 vs. D; d, *p* < 0.05 vs. C; e, *p* < 0.01 vs. D.

When doxorubicin was combined with melatonin, only nine genes were upregulated and the number of downregulated genes increased to 26 (Table 1). Sixteen genes were selected for further validation by performing specific real time RT-PCR analysis, establishing as selection criteria an expression level change of at least 1.5-fold either with doxorubicin alone or in combination with melatonin compared to vehicle-treated (control) cells. Thus, we were able to confirm (Figure 3A) that doxorubicin (1 µM) significantly induced the expression levels of *PTEN* (phosphatase and tensin homolog), *COP1* (constitutive photomorphogenic 1) and *CDKN1A* (cyclin-dependent kinase inhibitor 1A). These three genes are known to act as tumor supressors in breast cancer, and their mutations can cause chemoresistance [25,26,27]. Combination with melatonin (1 nM) further enhanced the anthracyclin stimulatory effect. Importantly, melatonin counteracted the stimulatory effect of doxorubicin over the expression of *NME1* (a supressor or metastasis in breast carcinoma) [28]; *MUC1* (a p53 supressor) [29]; *SNAI2* (involved in invasion and EMT) [30]; *BIRC5* (involved in doxorubicin resistance) [31]; and *TWIST1* (elevated in invasive breast cancer and involved in EMT [32,33] (Figure 3A). 

In some other cases, melatonin did not significantly change the expression levels of doxorubicin inducers (*BAX2*, *TP53*). *BAX2* and *TP53* are largely described as apoptosis inducers [34]. Finally, the pineal hormone further enhanced the inhibition of gene expression induced by doxorubicin, as in the case of *XBP1* (a promoter of tumor invasion) [35]; *WEE1* (involved in checkpoint progression) [36]; *GATA3* (highly expressed in estrogen dependent breast cancers) [37]; *PGR*, that usually is overexpressed in estrogen positive breast carcinomas [38] and *BCL-2*, a pro-survival protein whose targeting enhaces vulnerability to therapy in ER-positive breast cancer [39]. The expression of the oncogene *c-MYC* [40] was slightly downregulated by doxorubicin and dramatically inhibited by melatonin co-treatment (Figure 3A). 

The effect of combined treatment with doxorubicin and melatonin on gene expression was also tested in the triple negative hormone-independent MDA-MB-231 cell line. The expression of *TWIST1* and *CDKN1A* genes were prioritized for analysis, since the expression levels of these two genes were found to dramatically change and be highly modulated by melatonin in MCF-7 cells. An increase on the expression of *CDKN1A* was obtained after treatment with the anthracyclin; however, the expression of *TWIST1* was not stimulated by doxorubicin in this cell line. The addition of melatonin and doxorubicin together seem to cooperate to reach an additive effect on *TWIST1* inhibition in MDA-MB-231, although the inhibition reached after addition of melatonin is not as (Figure 3B). 

### 2.4. TWIST1 Protein Levels Are Upregulated by Doxorubicin and Inhibited by Melatonin in MCF-7 Cells

**Figure 4:**
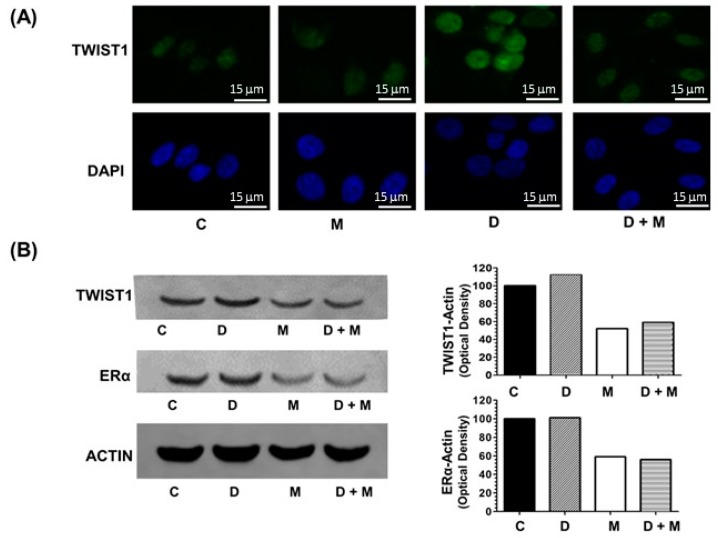
(**A**) TWIST1 detection by immunofluorescence (IF). MCF-7 cells treated for 12 h with doxorubicin (1 μM) and/or melatonin (1 nM) were fixed with formaldehyde and analyzed by IF microscopy after staining with anti-TWIST1 antibody and DAPI (4′6-diamidino-2-phenylindole). (**B**) Western blotting detection of TWIST1 and ERα in MCF-7 cells treated for 12 h with doxorubicin (1 μM) and/or melatonin (1 nM). B-actin was used as loading control. Scale bar: 15 µm.

As shown in Figure 3A, doxorubicin treatment induced a potent stimulation of *TWIST1* expression whereas co-treatment with melatonin counteracts this effect. For this reason, the protein levels of TWIST1 were determined by confocal microscopy and immunoblotting. As seen in Figure 4A, TWIST1 protein expression increased upon treatment with doxorubicin; however, melatonin alone or combined with doxorubicin decreased TWIST1 levels. We also tested the levels of ERα (Estrogen Receptor alpha), since it is known that: melatonin downregulates ER in MCF-7 cells [41], doxorubicin upregulates ER [42], and high levels of TWIST1 correlate with low levels of ER [43]. We found that the levels of ERα were also downregulated by melatonin (Figure 4B). Therefore, analogous regulation of TWIST1 expression by doxorubicin and melatonin was observed at both mRNA and protein levels in MCF-7 cells, which seem to be associated to ER expression (the whole western blots are available in supplementry data, Figure S1; the quantification of the density of the bands can be seen in supplementary data, Figure S3).

### 2.5. TWIST1 Gene Expression Is Regulated by Co-Culture of MCF-7 with Adipocytes

**Figure 5:**
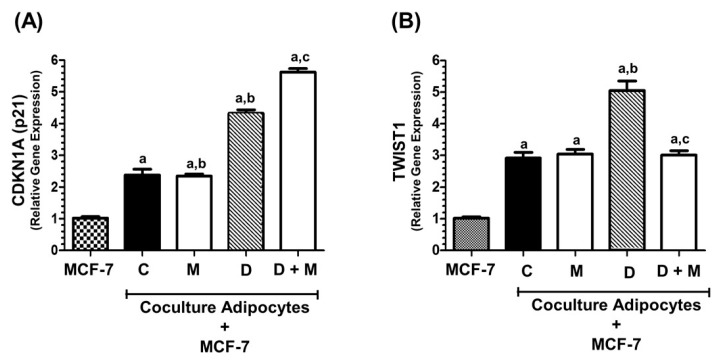
Effects of doxorubicin and/or melatonin on *CDKN1A* and *TWIST1* mRNA expression in MCF-7 cells co-cultured with mature adipocytes. Confluent human breast pre-adipocytes were differentiated in adipocyte differentiation media. MCF-7 cells and differentiated adipocytes were co-cultured as described in Materials and Methods, in the presence or absence of melatonin (1 nM) and/or doxorubicin (1 μM) or vehicle (ethanol at a final concentration lower than 0.0001%). Control MCF-7 cells were grown in the absence of adipocytes (column designated as MCF-7). After 24 h, total RNA was isolated from MCF-7 cells, reverse transcribed and cDNAs were used for analysis of (**A**) *CDKN1A* and (**B**) *TWIST1* mRNA expression by real time RT-PCR. All data are expressed as fold change relative to control cells (mean ± SEM) from three independent experiments. a, *p* < 0.001 vs. A; b, *p* < 0.001 vs. C; c, *p* < 0.001 vs. D.

Adipocytes induce EMT in breast cancer cells, and TWIST1 upregulation among other factors. On this basis, we examined whether adipocytes may induce the expression of *TWIST1* in breast cancer cells. The expression levels of CDKN1A gene were stimulated by adipocytes co-culture, potentiated by doxorubicin and further increased by melatonin (Figure 5A). Co-culture of MCF-7 cells with adipocytes increased the expresion levels of the *TWIST1* gene, and doxorubicin treatment further enhanced *TWIST1* levels. Co-treatment with melatonin diminished doxorubicin-induced expression of *TWIST1* (Figure 5B). 

### 2.6. Effects of Doxorubicin and Melatonin on the Expression of Angiogenesis-Related Genes 

We used the human angiogenesis RT² Profiler PCR Array, to determine the gene expression changes in MCF-7 cells upon treatment with doxorubicin (1 µM) either alone or in combination with melatonin (1 nM). The angiogenesis RT² Profiler PCR Array allows the simultaneous analysis of 84 genes involved in different processes linked to angiogenesis.

**Figure 6:**
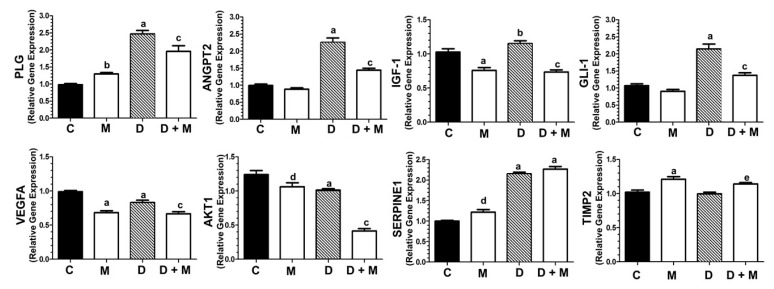
Gene expression profile of angiogenesis related genes. Effect of doxorubicin (1 µM) and/or melatonin (1 nM) on the gene expression in MCF-7 cells of the genes selected for analysis by specific RT-PCR: *PLG, ANGPT2, IGF1, GLI1, VEGFA, AKT1, SERPINE1* and *TIMP2*. All data are expressed as fold changes relative to control cells (mean ± SEM) from three independent experiments. a, *p* < 0.001 vs. C; b, *p* < 0.001 vs. C; c, *p* < 0.001 vs. D; d, *p* < 0.01 vs. C; e, *p* < 0.01 vs. D.

Doxorubicin alone upregulated the expression of 16 genes and downregulated eight genes involved in different aspects of angiogenesis (e.g., cell adhesion regulation, matrix degradation or growth factors) (Table 2). When both compounds were combined, the number of upregulated genes was reduced to five, whereas 14 genes were downregulated. To further validate these data, eight genes were selected (>1.5-fold change) and specifically analyzed by real time RT-PCR. Doxorubicin (1 µM) was found to significantly stimulate the expression of *PLG* (plasminogen, downregulated by Twist1 inhibitors [44]; *ANGPT2* (involved in breast cancer metastasis in brain) [45]; *IGF-1* (a stimulator of TWIST1) [46] and *GLI1* (activated upon TWIST1 activation) [47]. Co-treatment with melatonin (1 nM) blocked, at least partially, the stimulatory effect of the anthracyclin (Figure 6). The expression of *VEGFA* (inducer of angiogenesis) [48] and *AKT1* (inducer of TWIST1 activation) [49] was also significantly reduced by combined treatment with doxorubicin and melatonin, while *SERPINE1* expression was unaffected by melatonin. Finally, the expression of the protease inhibitor *TIMP2* was independent of doxorubicin, but induced by melatonin (Figure 6).

### 2.7. Effects of Doxorubicin and Melatonin on the Expression of Clock Genes 

**Figure 7:**
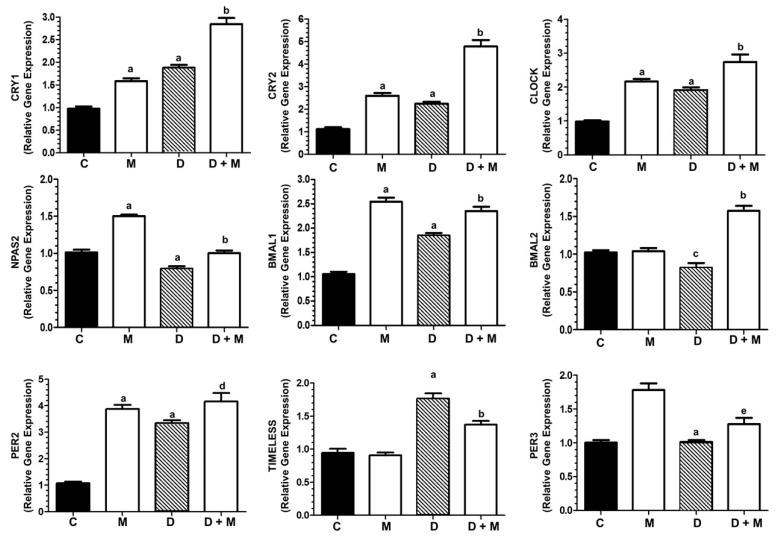
Expression of CLOCK genes. CLOCK genes expression changes upon treatment with doxorubicin (1 µM) and/or melatonin (1 nM) in MCF-7 cells. The following clock genes were selected for analysis by specific RT-PCR: *CRY1, CRY2, CLOCK, NPas2, BMAL1, BMAL2, PER2, TIMELESS* and *PER3*. All data are expressed as fold changes relative to control cells (mean ± SEM) from three independent experiments. a, *p* < 0.001 vs. C; b, *p* < 0.001 vs. D; c, *p* < 0.01 vs. C; d, *p* < 0.01 vs. D; e, *p* < 0.05 vs. D.

Since the inactivation of *CLOCK* genes seems to be a mechanism involved in breast cancer risk [50], we next assessed the effect of doxorubicin and melatonin on the expression of nine genes components of the circadian clock system. Doxorubicin alone enhanced the transcription of six genes and melatonin alone stimulated the expression of seven genes. The combination of both compounds remarkably enhanced the expression of *CRY1, CRY2, CLOCK, NPas2, BMAL1, BMAL2* and *PER2*. Only *TIMELESS* expression was enhanced by doxorubicin whereas the stimulatory effect of the anthracycline was partially counteracted by melatonin. *PER3* expression was independent of doxorubicin and stimulated by the presence of melatonin (Figure 7).

### 2.8. Combination of Melatonin and Doxorubicin on Kinase Intracellular Regulators

Next, we investigated the effect of treatment with doxorubicin and melatonin of MCF-7 cells on the activation/inactivation status of protein kinases by phosphorylation. This was accomplished by using the Proteome Profiler^TM^ Array (R&D Systems, Minneapolis, MN, USA) to test the level of phosphorylation of multiple protein kinases involved in different intracellular signaling pathways. 

**Figure 8:**
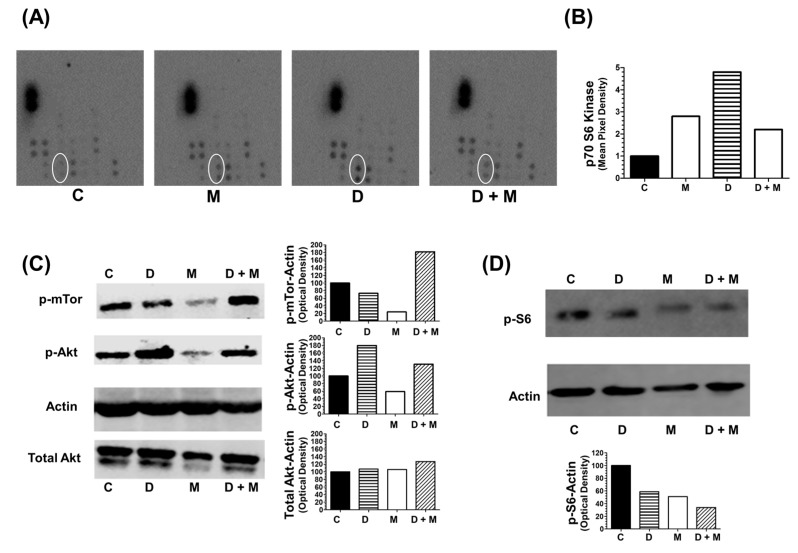
State of phosphorylation of protein kinases. (**A**) Representative images of phospho-kinase array blots assayed with protein extracts from MCF-7 cells, indicative the state of phosphorylation of multiple kinases was assayed using the Proteome Profiler^TM^ Array and protein extracts fromMCF-7 cells treated for 6 h with doxorubicin (1 μM) and/or melatonin (1 nM). The white oval remarks the dots corresponding to p70S6K levels. (**B**) The intensity of the signal of the dots corresponding to p70S6K was quantified by densitometry and expressed relative to the control. (**C**) Western blotting detection of phosphorylated mTOR (S2448), total Akt and phosphorylated Akt (S473) in MCF-7 cells treated for 6 h with doxorubicin (1 μM) and/or melatonin (1 nM). (**D**) Western blotting detection of S6 phosphorylation (S240/S244) in MCF-7 cells treated for 6 h with doxorubicin (1 μM) and/or melatonin (1 nM).

As shown in Figure 8A, doxorubicin induced the phosphorylation levels of p70S6 Kinase and this effect was counteracted by co-treatment with melatonin. The level of p70S6 Kinase phosphorylation relative to vehicle-treated control cells is represented in Figure 8B. The state of phosphorylation of Akt (S473) and mTOR (S2448) (as upstream regulators of p70S6 kinase) and the ribosomal protein S6 (S240) (as downstream target) were analyzed by western-blot. Melatonin alone reduced p-mTOR and both total and p-Akt levels. The levels of p-Akt (Ser 473) and total Akt were lower in MCF-7 cells treated with doxorubicin plus melatonin in comparison with cells treated only with doxorubicin (Figure 8C). The levels of p-S6 also decreased by melatonin treatment either alone or administered with doxorubicin (Figure 8D). (The whole western blots are available in supplementry data, Figure S2; Quantification of the density of the bands is available in supplementary data, Figures S4 and S5).

### 2.9. Doxorubicin and Melatonin Modulate the Expression of miR-34a, miR-29a, miR-31, miR-10a, miR145 and miR-10b in MCF-7 Cells

**Figure 9:**
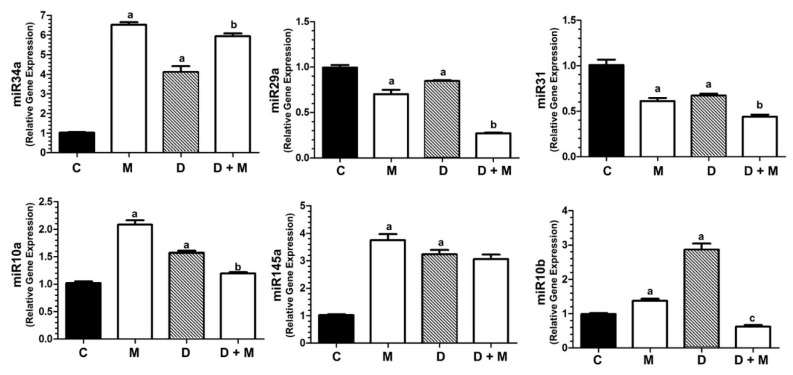
RT-PCR detection of miR-34a, miR-29a, miR-31, miR-10a, miR-145 and miR-10b. miRNA levels were analyzed in MCF-7 cells treated with doxorubicin alone (1 μM) and/or melatonin (1 nM) or vehicle (ethanol at a final concentration lower than 0.0001%). All data are expressed as fold change relative to control cells (mean ± SEM) from three independent experiments. a, *p* < 0.001 vs. C; b, *p* < 0.001 vs. D; c, *p* < 0.01 vs. C.

MicroRNAs can function either as oncogenes or tumor supressors by regulating intracellular signaling pathways [51]. Therefore, we decided to test the effect of doxorubicin and melatonin on the expression of various miRNAs involved in epithelial to mesenchimal transition (EMT), cell growth, migration, invasion, stem cell expansion and breast cancer progression, in correlation or not with TWIST1. As shown in Figure 9, the expression of miR-34a was stimulated by doxorubicin and further increased by melatonin co-treatment. miR-10b levels were also robustly induced by doxorubicin, although completely reduced by melatonin co-treatment. Contrasting this, miR-29a expression and miR-31 levels diminished by doxorubicin and melatonin co-treatment. The expression of miR-145, stimulated by doxorubicin, was not altered by melatonin co-treatment. The anthracyclin induced the expression of miR-10a, and melatonin significantly reduced this effect.

### 2.10. TWIST1 Blockade Abolishes the Stimulatory Effect of Doxorubicin on the Expression of miR-10b, miR-10a and VEGFA

**Figure 10:**
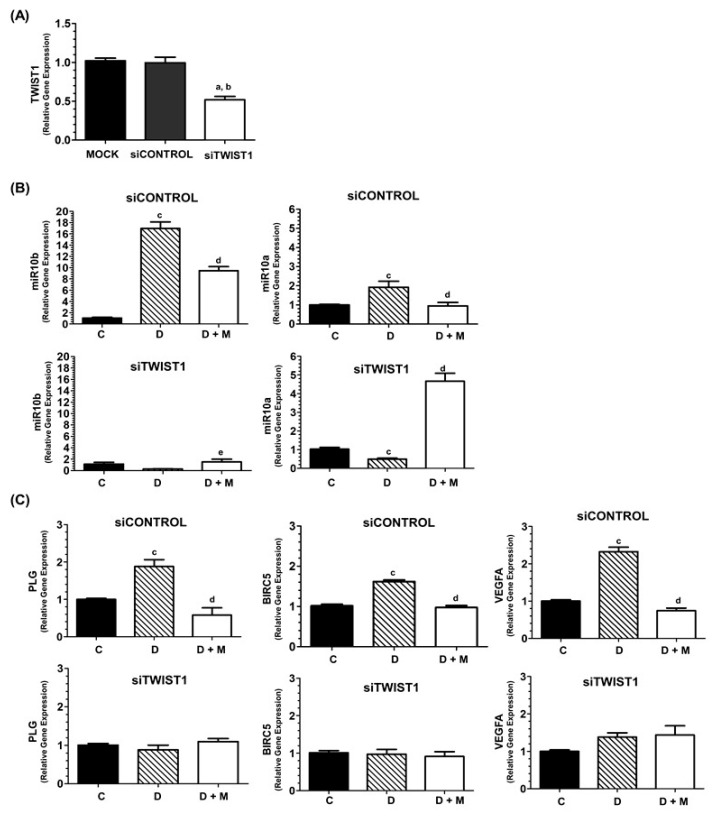
(**A**) RT-PCR detection of TWIST1 levels in MCF-7 cells transfected in the absence of siRNA (mock column), siControl or specific siTWIST1. (**B**) RT-PCR detection of miR-10b and miR-10a in MCF-7 cells transfected with siControl or siTWIST1. (**C**) RT-PCR detection of *PLG, BIRC5* and *VEGFA* levels in MCF-7 cells transfected with siControl or siTWIST1. After 72 h transfection, cells were treated for 6h with doxorubicin alone (1 μM) and doxorubicin plus melatonin (1 nM) or vehicle (ethanol at a final concentration lower than 0.0001%). All data are expressed as fold change relative to control cells (mean ± SEM) from three independent experiments. a, *p* < 0.001 vs. mock; b, *p* < 0.0001 vs. siControl; c, *p* < 0.001 vs. C; d, *p* < 0.001 vs. doxorubicin.

We next investigated the effect of knocking down *TWIST1* in MCF-7 cells. For this purpose, MCF-7 cells were transfected with either siCONTROL or siTWIST1, and expression of miR-10b, miR-10a and *VEGFA* was determined. As seen in Figure 10, the blockade of *TWIST1* completely abolished the strong induction of miR-10b, and also significantly diminished the induction of miR-10a by doxorubicin. In addition, the expression levels of *PLG, BIRC5* and *VEGFA* were significantly induced by doxorubicin in siControl-transfected MCF-7 cells and dramatically reduced by melatonin cotreatment; however, the expression changes observed upon doxorrubicin and combination treatment with melatonin were completely abolished in siTWIST1-transfected cells. These data indicate that these expression changes caused by treatment with doxorrubicin and melatonin are at least partially mediated by TWIST1. 

## 3. Discussion

The American Cancer Society reports that more than 70% of breast cancers are, at their initial stages, hormone-dependent [52], meaning that their growth is promoted by estrogens. When the tumor spreads out the breast, chemotherapy is a therapeutic option. Anthracyclines such as doxorubicin are amongst the most common drugs used for adjuvant and neoadjuvant chemotherapy. Doxorubicin binds to DNA in an intercalation process forming a stable complex [53]. Chemotherapy treatments provoke undesirable severe side effects and development of acquired chemoresistance, involving a variety of molecular events still not fully elucidated [54]. Thus, the use of adjuvant compounds might help to overcome resistance, to diminish side effects, and to improve drug tolerance. Although many of the gene expression and post-translational modifications triggered by chemotherapeutic drugs have been characterized [7,8,55], there are many molecular modifications triggered in tumor cells in response to chemotherapy remaining largely unknown. 

Melatonin behaves as a protective agent against doxorubicin-induced toxicity [56,57], enhancing apoptosis and cytotoxicity in cancer cells [19,58,59]. In rats, melatonin sensitizes mammary tumors to doxorubicin [18]; disruption of melatonin nocturnal production lead to a complete loss of tumor sensitivity to doxorubicin [17]. Yet most of the molecular mechanisms triggered by doxorubicin and modulated by melatonin remain unknown. As we have previously done for docetaxel [15], the aim of this work was to deepen into the knowledge of the molecular mechanisms triggered by doxorubicin and modified by the combination of doxorubicin and melatonin in breast cancer cells. 

First, we tested the effect of doxorubicin and melatonin on the proliferation, migration and invasion of MCF-7 and MDA-MB-231 cells. Melatonin potentiated the inhibitory effect of the anthracycline on MCF-7 proliferation, inhibited migration (independent of doxorubicin) and the invasive potential in MCF-7 cells, counteracting the stimulatory effect of the anthracycline, but not in MDA-MB-231 cells, indicating that the effectiveness of melatonin may be related to the estrogen receptor status. 

When we examined the gene expression profile of breast cancer related genes in MCF-7 cells, we found that melatonin modulated some of the changes induced by doxorubicin. We further analyzed the expression of 16 genes by specific RT-PCR. In some cases, melatonin and doxorubicin cooperated to stimulate gene expression: *PTEN*, a tumor suppressor gene whose mutations (particularly in breast cancer) cause chemoresistance [25]. *CDKN1A* (p21), the tumor suppressor controlling cell cycle and tumor progression, although, recently, it has been described that overexpression and an abnormal subcellular localization of p21 might correlate with poor response to chemotherapy [26]. *COP1*, another factor with a controversial effect in cancer, since it has been described both as involved in resistance to doxorubicin in leukemia cells [60] and as a suppressor of breast cancer progression [27].

Not all the tumor suppressor genes were modulated by melatonin; thus, *NME1*, a suppressor of metastasis in breast carcinoma [28], was stimulated by doxorubicin and melatonin had no effect on its expression. 

Melatonin significantly counteracted the stimulatory effect of doxorubicin on the expression of some genes: *MUC1*, (a p53 repressor) overexpressed in cancer [29], *SNAI2*, involved in invasion and epithelial to mesenchymal transition [30] and *BIRC5*, an inhibitor of apoptosis highly expressed in doxorubicin-resistant cells [31]. The regulation of *TWIST1* was the most striking result. TWIST1 is a transcription factor involved in mesoderm development [61,62]. *TWIST1* is re-expressed in cancer, since it bypasses p53-induced growth arrest [63], is elevated in invasive breast cancer [32], involved in EMT [20,21], and correlates with poor survival [22]. We found that treatment of MCF-7 cells with doxorubicin resulted in a strong induction of *TWIST1* expression and co-treatment with melatonin completely abolished this effect. The protein levels of TWIST1 were increased in doxorubicin treated cells and melatonin impaired this stimulatory effect. Our results suggest that doxorubicin has a dual action: it kills cancer cells, but simultaneously induces *TWIST1* expression leading to EMT of the surviving cells. The effect of doxorubicin on TWIST1 in MCF-7 cells was not obtained in MDA-MB-231 cells, again suggesting a correlation between melatonin action and the estrogen receptor status.

TWIST1 induces angiogenesis in vivo [64,65]. This prompted us to examine the gene expression profile of angiogenesis related genes in MCF-7 cells. Doxorubicin stimulated the expression of plasminogen (*PLG*), downregulated by compounds able to supress the TWIST1-dependent EMT [44]. Angiopoietin 2 (*ANGPT2*) involved in blood-brain barrier disruption and breast cancer metastasis in brain [45]. Insulin like growth factor 1 (*IGF1*) a positive regulator of TWIST1 [46]. *GLI1* (GLI family zinc finger 1), activated when the hedgehog signaling and TWIST1 are activated, leading to migration and invasion [47,66]. Importantly, melatonin partially blocked the stimulatory effect of doxorubicin on *PLG, ANGPT2, IGF-1* and *GLI1* expression indicating an anti-angiogenic effect of the indoleamine.

Dim light exposure at night induces a circadian disruption of nocturnal melatonin, thus contributing to doxorubicin resistance in breast cancer [17]. Since both melatonin and TWIST1 influence the expression of CLOCK-related genes [50,67,68], and inactivation of *CLOCK*, *CRY1*, *BMAL1*, and *PER1* has been proposed as a mechanism increasing breast cancer risk in shift workers [69], we next studied the effect of doxorubicin in the expression of CLOCK genes in MCF-7 cells. Doxorubicin stimulated *CRY1, CRY2, CLOCK, BMAL1, BMAL2* and *PER2* and melatonin significantly enhanced this stimulatory effect. The only exception was *TIMELESS*, induced by doxorubicin and downregulated when melatonin is included in the treatment. This might be another important finding of this work, since TIMELESS activates MYC, enhances ERα-mediated transactivation and contributes to tamoxifen resistance [70,71]. 

Moreover, we found that melatonin reduces the levels of *VEGFA*. This growth factor is upregulated by TWIST1, thus inducing angiogenesis in human umbilical vein endothelial cells [48], result in agreement with previous work [72]. *AKT1* was also significantly downregulated by melatonin, as previously described [49,73,74]. In chemoresistant ovarian cancer cells, inhibition of Akt phosphorylation, resulted in p70S6K inactivation and TWIST1 downregulation [75]. Therefore, we focused our attention on the intracellular pathways triggered by protein kinases phosphorylation. In a proteome profiler assay, we found that doxorubicin treatment induced a modest effect of melatonin on Akt phosphorylation at Ser473. Phosphorylation of Akt correlates with poor prognosis and has been found in MCF-7 cells but not in MDA-MB-231 [76,77]. Suppression of PTEN activates the Akt/mTOR/p70S6K signaling and decreases sensitivity of MCF-7 cells to doxorubicin [78]. p-Akt phosphorylates TWIST1 thus contributing to EMT and breast cancer metastasis [49]. In our hands, melatonin downregulates p-mTOR and both total and p-Akt. However, we did not observe significant changes in the levels of p-mTOR after treatment with doxorubicin or co-treatment with melatonin; the modest inhibition of p-Akt in the cells treated simultaneously with both compounds suggests that TWIST1 activation by doxorubicin might respond to other signaling pathways apart from Akt/mTOR. We also observed an increase in the phosphorylation of P70S6K in response to doxorubicin. In head and neck squamous cell carcinoma, activation of the S6 pathway has been correlated with EMT induction and metastasis [79], and poor prognosis and tamoxifen resistance in breast cancer [80]. Interestingly, our results showed that the phosphorylation of S6 induced by doxorubicin is inhibited by melatonin, in parallel with a decrease in the levels of ERα. We tested ERα levels in parallel to TWIST levels, since it has been previously described that melatonin downregulates ER in MCF-7 cells [41]. Doxorubicin upregulates ER [42] and cell lines with high levels of TWIST1 have low levels of ER, being an increase of TWIST1 levels a mechanism that might contribute to the generation of hormone-resistant ER-negative breast cancer [43]. In our model, melatonin treatment decreased the levels of both proteins. 

We next determined the expression of *TWIST1* in MCF-7 cells co-cultured with adipocytes. Adipocytes induce EMT in breast cancer cells by increasing, among some other factors, the expression of *TWIST1* [81,82]. Since transcriptional activation of the *CDKN1A* promoter by p53 is significantly repressed by induction of *TWIST1* [83], we tested whether adipocytes can induce the expression of *TWIST1* in MCF-7 cells. Indeed, co-culturing MCF-7 cells with adipocytes, even in absence of any treatment, increased the expresion of *TWIST1*. This effect was enhanced by doxorubicin. Co-treatment with melatonin abolished the stimulatory action of doxorubicin over *TWIST1* expression. The expression of *CDKN1A* was also stimulated by the presence of adipocytes in the co-cultures and was upregulated by doxorubicin. In this case, cotreatment with doxorubicin plus melatonin further increased the levels of *CDKN1A*. Therefore, adipocytes promote breast cancer cells adquisition of a more aggressive phenotype and doxorubicin treatment contributes to stimulate the EMT processes, whereas melatonin, at physiological concentrations, protects against TWIST1-mediated EMT.

MicroRNAs have been implicated in a wide range of intracellular signaling pathways [84]. Concerning carcinogenesis, they can act as oncogenes or tumor supressor genes [51]. In the last set of experiments of this work, we tested the expression of some microRNAs known to play different roles in cancer, directly or indirectly related to TWIST1.

MicroRNA-34a protects against breast cancer proliferation, invasion and metastasis by directly targeting TWIST1 [85,86,87]. In MCF-7 cells, doxorubicin stimulates the expression of miR-34a, perhaps a mechanism trying to compensate *TWIST1* induction. Importantly, melatonin treatment further stimulated the expression of miR-34a. MicroRNA-29a has been involved in MCF-7 cell proliferation and invasion dependent on the IGF pathway [88]. Downregulation of miR-29a correlates with p21 and p53 upregulation, leading cancer cells to apoptosis [89]. We found that cells treated with both doxorubicin plus melatonin showed lower levels of miR-29a, what might be another protective mechanism against malignity. The role of miR-31 in breast cancer is controversial. Whilst some reports conclude thart miR-31 inhibits invasion and metastasis in breast cancer cell line models [90], other authors claim that miR-31 promote cell proliferation and metastasis [91]. In our hands, miR-31 levels are downregulated by doxorubicin and co-treatment with melatonin further inhibited miR-31 expression. 

MicroRNA-10a is an inhibitor of breast cancer progression, promoting apoptosis in MCF-7 cells, via PI3K/Akt/mTOR. Upregulation of miR-10a also suppresses the activation of p70S6K [92]. Our results showed that, although melatonin alone has a stimulatory effect on its expression, is not able to enhance the modest induction of miR-10a in response to doxorubicin, suggesting that miR-10a does not play a crucial role in *TWIST1* induction by doxorubicin. 

miR-145 provides a link between p70S6K to TWIST1. P70S6K prevents the maturation of miR-145, and consequently, *TWIST1* is upregulated, leading to metastasis [93]. Reactivation of miR145 supressed migration and invasion of breast cancer cells [94]. In our model, the expression of miR-145 is stimulated by doxorubicin and melatonin, however, the addition of both compounds simultaneously does not further increase miR-145 levels.

We also tested the levels of miR-10b, described as an oncogenic microRNA involved in tumor invasion, angiogenesis, cellular motility and metastasis [95,96]. TWIST1 induces miR-10b [23,96] promoting breast cancer bone metastasis [97]. We found that doxorubicin strongly stimulates miR-10b expression, whereas melatonin concomitantly added with the anthracyclin abolishes this stimulatory effect. Thus, the inhibition of miR-10b, probably a consequence of *TWIST* inhibition, might be another mechanism participating in the protective role of melatonin on doxorubicin breast cancer treated cells. When we were preparing our manuscript, Chao et al. reported that melatonin supresses lung cancer metastasis, preventing EMT by downregulating *TWIST1*, which agrees with our results, indicating that *TWIST1* might be a novel target of melatonin in different cancer types [98].

Importantly, the blockade of *TWIST1* expression by siRNA technology completely abolished the induction of miR-10b, *PLG* and *BIRC5* by doxorubicin, allows a higher effect of melatonin on miR-10a expression and partially impaired the stimulatory action of doxorubicin on *VEGFA* expression. These data indicate that TWIST could be involved in the expression changes of these genes and miRNAs observed upon treatment with doxorrubicin and melatonin.

## 4. Materials and Methods

### 4.1. Cell Lines and Culture Conditions 

Human breast cancer cell lines MCF-7 (hormone-dependent, ERα-positive) and MDA-MB-231 (hormone-independent, triple negative) were acquired from the American Tissue Culture Collection (Rockville, MD, USA). They were maintained as monolayer cultures in 75 cm^2^ plastic culture flasks in Dulbecco’s Modified Eagle’s Medium (DMEM) (Sigma-Aldrich, Madrid, Spain) supplemented with 10% fetal bovine serum (FBS) (PAA Laboratories, Pasching, Austria), 2 mM L-Glutamine (GIBCO, Langley, OK, USA), penicillin (20 units/mL) and streptomycin (20 μg/mL) (Sigma-Aldrich, Madrid, Spain) at 37 °C in a humid atmosphere containing 5% CO_2_.

Breast preadipocytes (ZenBio, RTP, Durham, NC, USA) were plated in six-well TC plates (Corning, Lowell, MA, USA), and propagated in preadipocyte media (ZenBio, RTP, Durham, NC, USA) at 37 °C in a humidified atmosphere of 5% CO_2_.

### 4.2. Co-Culture of Breast Adipocytes and MCF-7 Cells as an In Vitro Breast Cancer Model 

Confluent pre-adipocytes were propagated in adipocyte differentiation media (ZenBio, RTP, Durham, NC, USA) for 7 days. Following the differentiation media, the resulting adipocytes were propagated in DMEM:F12 (GIBCO^®^, Invitrogen, Carlsbad, CA, USA) containing 1% PSF (GIBCO^®^, Invitrogen), 10%fetal bovine serum (Sigma, St. Louis, MO, USA), and 5 ng/mL insulin (GIBCO^®^, Invitrogen) for 7 days.

Mature adipocytes and MCF-7 cells were co-cultured together using Falcon 24-multiwell plates and Falcon cell culture inserts. First, mature adipocytes (12 × 10^3^ cells/well) were cultured in the upper chamber in DMEM supplemented with 10% FBS for 24 h. At this time, inserts containing MCF-7 cells (5 × 10^3^ cells/well) were seeded in the lower chamber and co-cultured with or without mature adipocytes (12 × 10^3^ cells/well) in fresh DMEM medium containing melatonin (1 nM) and/or doxorubicin (1 μM) or vehicle (ethanol at a final concentration lower than 0.0001%). After 24 h, total RNA was isolated from MCF-7 cells and purified with the Nucleospin RNA II kit (Macherey-Nagel, GmbH & Co. Düren, Germany), and used for cDNA synthesis.

### 4.3. Cell Proliferation Assay

MDA-MB-231 and MCF-7 cells were cultured for 3 days in media containing melatonin (1 nM) and/or doxorubicin (ranging from 0.1 nM to 1 μM) or vehicle (ethanol at a final concentration lower than 0.0001%). Cell proliferation was measured by the MTT [3(4,5-dimethylthiazol-2-yl)-2,5-diphenyl tetrazolium bromide] method [99], reading absorbance at 570 nm in a microplate reader (Labsystems Multiskan RC 351, Vienna, VA, USA). MTT was obtained from Molecular Probes Inc (Molecular Probes, Eugene, OR, USA). Each result represents the media ± S.E.M of at least three independent experiments and data are presented as percentage of control untreated cells.

### 4.4. Cell Migration Assay: Wound Healing Assay

MDA-MB-231 and MCF-7 cells were seeded into 6-well plates (Life Sciences, Tewksbury, MA, USA) and cultured for 24 h in DMEM supplemented with 10% FBS (csFBS) to reach full confluence. A line of cells was scraped away in each plate using a pipette tip. Media were replaced by fresh ones containing melatonin (1 nM) and/or doxorubicin (1 μM) or vehicle (ethanol at a final concentration lower than 0.0001%). At least four randomly selected views along the scraped line in each plate were photographed using an ORCA R2 camera (Hamamatsu Photonics, Massy Cedex, France) attached to a microscope set Nikon Ti (Werfen Group, Barcelona, Spain) at 10× magnification. Microphotographs were taken every ten minutes during the course of the experiment, which was terminated after 24 h. Initial and final wound sizes were determined using the Nis Elements v.3.8 software (Nikon, Tokyo, Japan) and the difference between the two was used to determine migration distance as follows: initial wound size minus final wound size divided by two. Each assay was performed at least three times.

### 4.5. Three-Dimensional Spheroid Invasion Assays

MDA-MB-231 and MCF-7 cells were suspended in DMEM medium plus 5% methyl cellulose (Sigma-Aldrich, Madrid, Spain) at 80,000 cells/mL. Cell spheroids were subsequently formed by serial pippeting of 25 μL into a non-adhesive petri dish (2000 cells/spheroid) and incubated in an inverted position for 18 h. Next, each spheroid was transferred to an individual well of 96-well plate and embedded into a volume of 110 μL of 2.3 mg/mL bovine collagen type I matrix (PureCol, San Diego, CA, USA) from advanced Biomatrix, and filled with 100 μL of complete media. 

Collective cell invasion was monitored using a Zeiss Cell Observer Live Imaging microscope coupled with a CO_2_ and temperature maintenance system. Time-lapse images were acquired every 20 min during 24 h using a Zeiss AxioCam MRc camera (Carl Zeiss, Sliedrecht, The Netherlands). The area of each individual spheroid was measured using ImageJ Fiji analysis program (version 1.50i, Bethesda, MD, USA). The invasive area was determined by calculating the difference between the final area (at each represented time) and the initial area (*t* = 0 h), and data were normalized to the control (vehicle-treated) cells. Each assay was performed at least three times using quadruplicates.

### 4.6. RNA Isolation and cDNA Synthesis 

Total RNA was isolated from MCF-7 and MDA-MB-231 cells and purified with the Nucleospin RNA II kit (Macherey-Nagel, GmbH & Co. Düren, Germany) following the manufacturer’s instructions. The concentration and purity of RNA was quantified by measuring the absorbance in a spectrophotometer (Nanodrop, Thermofisher, Control Técnica, Boadilla del Monte, Spain). The absorbance ratio A260 nm/A280 nm was always > 1.9. For cDNA synthesis, 0.5 μg of total RNA was used as template using the RT^2^ First Strand Kit (Qiagen, Germantown, MD, USA), following the manufacturer´s instructions. First, the genomic DNA was eliminated by incubating the samples 5 min at 42 °C. After mixing with the reverse-transcription mix, the samples were incubated for exactly 15 min at 42 °C in a final volume of 20 μL. The reaction was stopped by incubating at 95 °C for 5 min. 91 μL of RNA-free water was added to each reaction and samples were kept on ice until proceeding with the real-time PCR protocol.

### 4.7. RT^2^ Profiler^TM^ PCR Array

Pathway-focused gene expression profiling was performed using a 96-well human breast cancer PCR array (RT^2^ Profiler PCR array-PAHS-131ZA, Human Breast Cancer PCR Array, Qiagen, Germantown, MD, USA) and a 96-well human angiogenesis PCR array (RT^2^ Profiler PCR array-PAHS-024ZA, Human Angiogenesis PCR Array, Qiagen, Germantown, MD, USA). In these arrays, each well contains all the components required and designed to generate single, gene-specific amplicons, testing the expression of 84 genes related to breast cancer pathways or to angiogenesis, 5 housekeeping genes and controls for data normalization, genomic DNA contamination detection, RNA sample quality and general PCR performance. 

Briefly, MCF-7 were treated for 6 h with doxorubicin (1 µM) alone or in combination with 1 nM melatonin and/or vehicle (ethanol at a final concentration lower than 0.0001%). RNA was extracted and reverse transcribed as explained in the Section 4.6. The cDNA template was mixed with the appropriate amount of RT^2^ SYBR Green qPCR Mastermix (Qiagen GmbH, Hilden, Germany), aliquoted 25 μL to each well of the same plate, and then the real-time PCR cycling program was performed in an MX3005P (Agilent, Santa Clara, CA, USA) following the manufacturer’s instructions. 

Cycling conditions were: 1 cycle at 95 °C for 10 min followed by 40 cycles using the following temperature profile: 95 °C for 30 sec (denaturation) and 60 °C for 60 sec (annealing/extension). Dissociation curves were performed to verify that only a single product was amplified. The Ct data for each gene were analyzed using the Qiagen RT^2^ profiler PCR array data analysis software. Data are represented as fold-regulation between the experimental groups and the control cells. 

### 4.8. Measurement of Specific mRNA Gene Expression 

Analysis of specific mRNA gene expression was carried out by RT-PCR after incubation of cells for 6 h with doxorubicin (1 µM) and/or melatonin (1 nM), or vehicle (ethanol at a final concentration lower than 0.0001%). Total cellular RNA was isolated, reverse transcribed and the RT-PCRs were performed using the same temperature profile described in Section 4.7. 

Reactions were run in triplicate and melting curves were performed to verify that only a single product was amplified. Each result represents the media of three to five independent experiments and data are presented as fold-change between the experimental groups and the control cells. The primers used for amplification (Sigma Genosys Ltd., Cambridge, UK) are listed in Table 3. 

### 4.9. Real-Time RT-PCR Data Analysis

For the primers used there were no differences between transcription efficiencies, and the fold-change in each sample was calculated by the 2^−∆∆Ct^ method [100]. The fractional cycle at which the amount of amplified target becomes significant (Ct) was automatically calculated by the PCR program. The relative expression of β-actin was used to normalize gene expression.

### 4.10. Quantification of miRNAs Expression 

Total RNA, including the small RNA fraction, was extracted from MCF-7 cells using TRIzol reagent (Invitrogen). TaqMan^®^ MicroRNA Reverse Transcription Kit (Life Technologies, Carlsbad, CA, USA) was used for the RT step. Corresponding TaqMan^®^ Human MicroRNA Assays (Life Technologies, Carlsbad, CA, USA) were used to determine the copy number of miR-10a, miR-10b, miR-29a, miR-31, miR-34a, miR-133a and miR-145. MiRNAs levels were normalized to the expression levels of RNU6B, which was measured in parallel for each specific miRNA determination. Each sample was assayed in triplicate and a negative control was included in each experiment. Cycling conditions were an initial cycle of 95 °C for 10 min, followed by 50 cycles of 95 °C for 15 s, and 60 °C for 90 s, performed in an MX3005P (Agilent, Santa Clara, CA, USA) thermocycler. 

### 4.11. Phosphokinase Screening

MCF-7 cells were initially seeded into 6-well plates at a density of 5 × 10^5^ cells per well in DMEM supplemented with 10% FBS and incubated at 37 °C for 24 h to allow for cell attachment. Then, media were replaced by fresh ones with 10% FBS and containing either doxorubicin alone (1 μL) and/or melatonin (1 nM) or vehicle (ethanol at a final concentration lower than 0.0001%). After 6 h of incubation, cells were washed with PBS, lysed and screened for phosphorylation of kinases with the Proteome Profiler^TM^ Array (Human Phospho-kinase Array Kit, Catalog Number ARY003B, R&D Systems, Minneapolis, MN, USA) according to manufacturer´s instructions.

### 4.12. Antibodies 

Antibody against actin (A5441) was purchased from Sigma Aldrich (Madrid, Spain). Antibodies against TWIST1 (2c1a, Cc-81417) and ER (F-10, Sc-8002) were purchased from Santa Cruz Biotechnology (Santa Cruz, CA, USA). Antibodies against phospho-S6 ribosomal protein (D68F8), Phospho-Akt (Ser473) (92712), Akt (4691L) and phospho-mTOR (Ser2448) (D9C2) were purchased from Cell Signaling (Danvers, MA, USA)

### 4.13. Western Blotting

MCF-7 cells were initially seeded into 6-well plates at a density of 5 × 10^5^ cells per well in DMEM supplemented with 10% FBS and incubated at 37 °C for 24 h to allow for cell attachment. Then, media were replaced by fresh ones with 10% FBS and containing either doxorubicin alone (1 μM) or in combination with melatonin (1 nM) and/or vehicle (ethanol at a final concentration lower than 0.0001%). Samples for Western blotting were prepared by harvesting cells under appropriate conditions for centrifugation, resuspending the pellet in SDS buffer, and boiling for 5 min. Equal amounts of protein lysate were resolved by SDS-PAGE, transferred to polyvinylidene difluoride (PVDF) membrane and blocked in a buffer containing 5% non-fat milk. Western blots were probed with various primary antibodies listed above. The blots were stripped and re-probed with anti-β-actin antibody (Sigma, St. Louis, MO, USA) to evaluate loading. Images were acquired with the Odyssey infrared imaging system and analyzed by the software program as specified in the Odyssey software manual.

### 4.14. Immunofluorescence Staining

For immunofluorescence assays, MCF-7 cells were seeded onto glass coverslips in 6-well plates and grown for 24 h. Then, media were replaced by fresh ones with 10% FBS and containing either doxorubicin alone (1 μM) and/or melatonin (1 nM), or vehicle (ethanol at a final concentration lower than 0.0001%). After 12 h, cells were washed with PBS, fixed in 4% formaldehyde solution for 30 min and then permeabilized with 0.2% Triton X-100/PBS for 15 min (twice). Coverslips were incubated with anti-TWIST1 primary antibodies overnight at 4 °C, followed by 1 h incubation with fluorescence-tagged secondary antibody at room temperature, and then stained with DAPI for 5 min. Finally, coverslips were observed, and cell images were captured at 40× magnification with an LSM-510 laser scanning microscope (Carl Zeiss Inc., Sliedrecht, The Netherlands) fluorescence microscope. 

### 4.15. Small Interfering RNA (siRNA)

Small interfering RNA (siRNA) technology was used to knock down TWIST1 expression. A mixture of four siRNA (ON-TARGETplus SMARTpool TWIST1 siRNA) were purchased from Dharmacon (Lafayette, CO, USA). The target sequences were: 5′-UGAGCAACAGCGAGGAAGA-3′, 5′-GGAGUCCGCAGUCUUACGA-3′, 5′-GCAAAUAGAUCCGGUGUCU-3′ and 5′-AAUCAGAGGAACUAUAAGA-3′. A non-silencing siRNA siGENOME RISC-Free Control was used as negative control. MCF-7 cells were transfected with pooled siRNA using Lipofectamine 2000 (Invitrogen). The group designed as Mock corresponded to MCF-7 cells going through the transfection process without addition of siRNA. After 72 h, cells were treated with doxorubicin and/or melatonin for 6 h and RNA was extracted using Trizol (Gibco). Gene expression and miRNAs expression were determined by real time RT-PCR, as described in Section 4.8 and Section 4.10.

### 4.16. Statistical Analysis

Statistical analyses were performed using GraphPad Prism software. The data are expressed as the means ± standard errors of the mean (SEM). Statistical differences between groups were analyzed using One-Way Analysis of Variance (ANOVA), followed by the Student-Newman-Keuls test. Results were considered as statistically significant at *p* < 0.05.

## 5. Conclusions

**Figure 11:**
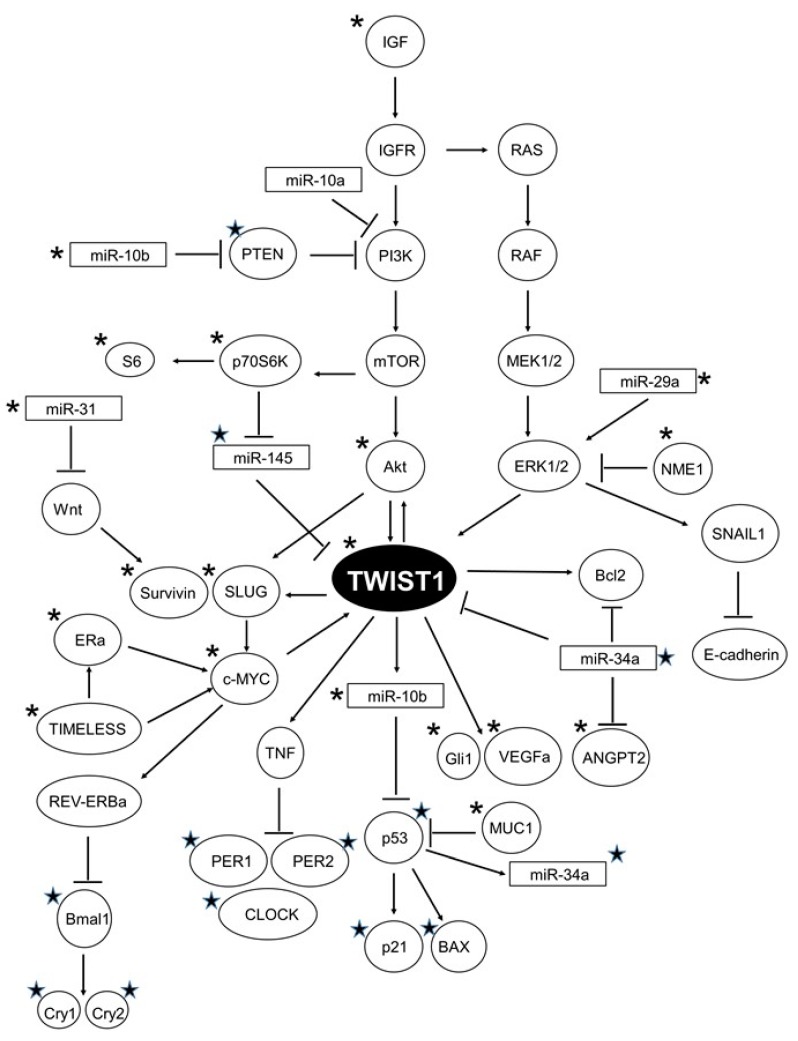
Proposed model of the pathways converging on TWIST1 that are altered by doxorubicin and modulated by melatonin. ★: Upregulated by melatonin; *: downregulated by melatonin.

In summary, this study has extensively contributed to deciphering multiple molecular changes modulated by doxorubicin and melatonin treatment in breast cancer cells that include gene expression changes, kinase activation and miRNAs expression. A model is proposed in Figure 11, indicating the factors induced or inhibited by melatonin. 

According to these original data, TWIST1 emerges as a central player in doxorubicin-treated breast cancer cells, since some of the upstream regulators of TWIST1 (PTEN, Akt, IGF, p70S6K, c-Myc, TIMELESS, miR-29a, miR-34a, miR145) and dowstream effectors (GLI1, Survivin, p21, Bcl-2, Bax, VEGFa, SLUG1, Per1, Per2, miR-10b) have all been found altered upon doxorubicin treatment. Importantly, melatonin may exert a protective role thereby modulating many of the undesirable effects of this chemotherapeutic agent, preventing cell proliferation, migration, invasion and EMT in ER-positive breast cancer cells.

## Figures and Tables

**Table 1:** The table summarizes the distribution of breast cancer gene categories induced or repressed in MCF-7 cells treated with doxorubicin (1 µM), doxorubicin plus melatonin (1 nM) or melatonin (1 nM) for 6 h. Pathway-focused gene expression profiling was performed using the Human Breast Cancer RT^2^ Profiler PCR Array. The number of up and downregulated genes in each category is indicated.

Breast Cancer Array	D vs. C		D + M vs. D		M vs. C	
	Up	Down	Up	Down	Up	Down
Angiogenesis, cell adhesion and proteases	5	3	2	1	0	2
Breast cancer classification markers	2	1	1	4	1	5
Signal transduction	8	3	3	5	3	6
Cell cycle	5	1	0	7	4	3
Apoptosis and DNA damage and repair	7	2	1	6	7	3
Transcription factors	0	7	2	3	2	3

D: Doxorubicin (1 µM), M: Melatonin (1 nM), C: control.

**Table 2:** The table summarizes the number of angiogenesis related genes which expression was induced or repressed in MCF-7 cells treated with doxorubicin (1 µM), doxorubicin plus melatonin (1 nM) or melatonin (1 nM) for 6 h. Genes were grouped as “adhesion molecules”, “proteases, inhibitors and matrix proteins”, “cytokines and other factors” and “growth factors and receptors”. Pathway-focused gene expression profiling was performed using the Human Angiogenesis RT^2^ Profiler PCR Array.

Angiogenesis Array	D vs. C		D + M vs. D		M vs. C	
	Up	Down	Up	Down	Up	Down
Adhesion molecules	4	0	0	2	0	1
Proteases, inhibitors and matrix proteins	4	2	1	5	2	1
Cytokines and other factors	0	3	1	3	0	0
Growth factors and receptors	8	3	3	4	2	0

**Table 3:** Primer sequences of genes tested by real time RT-PCR.

Gene	Forward	Reverse
*PTEN*	AGGTTTCCTCTGGTCCTGGT	CGACGGGAAGACAAGTTCAT
*COP1*	CTGGAAGCAGAATCACATGC	TGTGCTATCCTCACTGACAGG
*CDKN1A*	CAGCATGACAGATTTCTACC	CAGGGTATGTACATGAGGAG
*NME1*	CAGAAGTCTCCACGGATGGT	AGAAAGGATTCCGCCTTGTT
*MUC1*	GCAAGAGCACTCCATTCTCAATT	TGGCATCAGTCTTGGTGCTATG
*SNAI2*	CAGTGATTATTTCCCCGTATC	CCCCAAAGATGAGGAGTATC
*BIRC5*	TCTCCGCAGTTTCCTCAAAT	GGACCACCGCATCTCTACAT
*TWIST1*	CTAGATGTCATTGTTTCCAGAG	CCCTGTTTCTTTCAATTTGG
*BAX*	AACTGGACAGTAACATGGAG	TTGCTGGCAAAGTAGAAAAG
*TP53*	CCTATGCTTGTATGGCTAAC	TAGATCCATGCCTTCTTCTTC
*XBP1*	ACTGGGTCCAAGTTGTCCAG	TCACCCCTCCAGAACATCTC
*WEE1*	AGTGCCATTGCTGAAGGTCT	ACCTCGGATACCACAAGTGC
*GATA3*	CGGTCCAGCACAGGCAGGGAGT	GAGCCCACAGGCATTGCAGACA
*PGR*	GAGAGCTCATCAAGGCAATTGG	CACCATCCCTGCCAATATCTTG
*BCL2*	CCTTTGGAATGGAAGCTTAG	GAGGGAATGTTTTCTCCTTG
*c-MYC*	TGAGGAGGAACAAGAAGATG	ATCCAGACTCTGACCTTTTG
*PLG*	TAGATTCTCACCTGCTACAC	CGCAGTAGTCATATCTCTTTTC
*ANGPT2*	AAGAGAAAGATCAGCTACAGG	CCTTAGAGTTTGATGTGGAC
*IGF1*	ATAGAGCCTGCGCAATGGAA	GAGATGGGAGATGTTGAGAGCA
*GLI1*	CTCGTAGCTTTCATCAACT	TTTTTGGTGATTCATCTGGG
*VEGFA*	TGGTGATGTTGGACTCCTCA	GGGCAGAATCATCACGAAGT
*AKT1*	AAGTACTCTTTCCAGACCC	TTCTCCAGCTTGAGGTC
*SERPINE1*	ATCCACAGCTGTCATAGTC	CACTTGGCCCATGAAAAG
*TIMP2*	GGCCTGAGAAGGATATAGAG	CTTTCCTGCAATGAGATATTCC
*CRY1*	TTTTGCAGGGAAGCCTCTTA	CTGCTATTGCCCTGTTGGTT
*CRY2*	GCGAAAGCTGCTGGTAAATC	TACCTGCCCAAATTGAAAGC
*CLOCK*	TATCATGCGTGTCCGTTGTT	ACAAGGCATGTCCCAGTTTC
*NPAS2*	AAGGCTTCCAGTCTTGCTGA	CGGGACCAGTTCAATGTTCT
*BMAL1*	GACGAGGCAGCTGAGGTTAC	CCACAGCACAGGCTATTTGA
*BMAL2*	AATCCAACTGTGCACCATCA	GCTACCAGGCAAAACCAGAG
*PER2*	AGTGGGACTGGAAAATGCTG	CACACACAGAAGGAGGAGCA
*TIMELESS*	CACTGGCGTCATCATCAATC	GCAGGAGGAAGACAACTTGC
*PER3*	TCTTTGGGTCCAGTTGTTCC	TCCTGGCGTCTTCTCACTTT
*ACTIN*	TAGCACAGCCTGGATAGCAA	AAATCTGGCACCACACCTTC

## Supplementary Materials

The following are available online at https://www.mdpi.com/2072-6694/11/7/1011/s1, Figure S1: Western blot analysis of TWIST1, ER and actin, Figure S2: Western blot analysis of p-mTOR, Total Akt, p-Akt and actin, Figure S3: Western Cuantification of the density of the bands included in Figure 4. It was made by using the image proccessing package ImageJ Fiji, Figure S4: Western Cuantification of the density of the bands included in Figure 8C. It was made by using the image proccessing package ImageJ Fiji, Figure S5: Western Cuantification of the density of the bands included in Figure 8D. It was made by using the image proccessing package ImageJ Fiji.
