# Supplementary material for: Deciphering the Molecular Basis of Melatonin Protective Effects on Breast Cells Treated with Doxorubicin: TWIST1 a Transcription Factor Involved in EMT and Metastasis, a Novel Target of Melatonin
Source: Cancers (Basel). 2019 Jul 19;11(7):1011. doi: 10.3390/cancers11071011 (PMC6679136; doi:10.3390/cancers11071011)
Supplement: Supplementary file 1 [file cancers-11-01011-s001.pdf]

Supplementary Materials: Deciphering the Molecular Basis of Melatonin Protective Effects on Breast Cells Treated with Doxorubicin: TWIST1 a Transcription Factor Involved in EMT and Metastasis, a Novel Target of Melatonin

Javier Menéndez-Menéndez, Francisco Hermida-Prado, Rocío Granda-Díaz, Alicia González, Juana María García-Pedrero, Nagore Del-Río-Ibisate, Alicia González-González, Samuel Cos, Carolina Alonso-González and Carlos Martínez-Campa

Supplementary Methods: Western Blot analysis

Samples for Western blotting were prepared by harvesting cells under appropriate conditions for centrifugation, resuspending the pellet in SDS buffer, and boiling for 5 min. Equal amounts of protein lysate were resolved by SDS-PAGE, transferred to polyvinylidene difluoride (PVDF) membrane and blocked in a buffer containing 5% non-fat milk. Western blots were probed with various primary antibodies listed above. The blots were stripped and re-probed with anti-β-actin antibody (Sigma, St. Louis, MO) to evaluate loading. Images were acquired with the Odyssey infrared imaging system and analyzed by the software program as specified in the Odyssey software manual. When mambranes were used for more than one detection, membranes were sliced at the appropriated molecular weight size to incubate with the different antibodies. Cuantification of the density of the bands was made by using the image processing package ImageJ Fiji. Uncropped images of the immunoblots can be found in Supplementary Figures S1 (corresponding to Figure 4 of the manuscript) and Figure S2 (corresponding to Figure 8 of the article). The quantification of the bands can be found in Figures S3 (corresponding to Figure 4), S4 (corresponding to Figure 8C) and S5 (corresponding to Figure 8D).

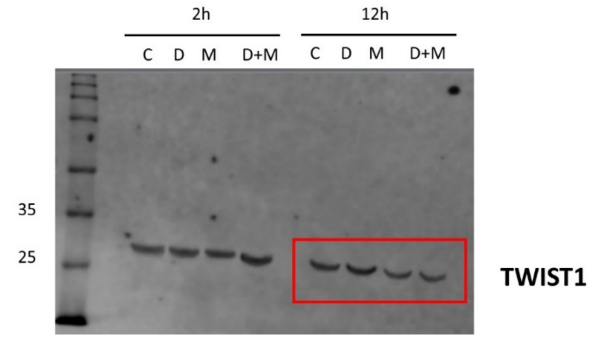

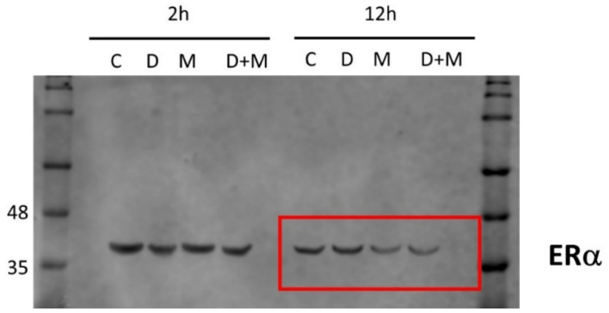

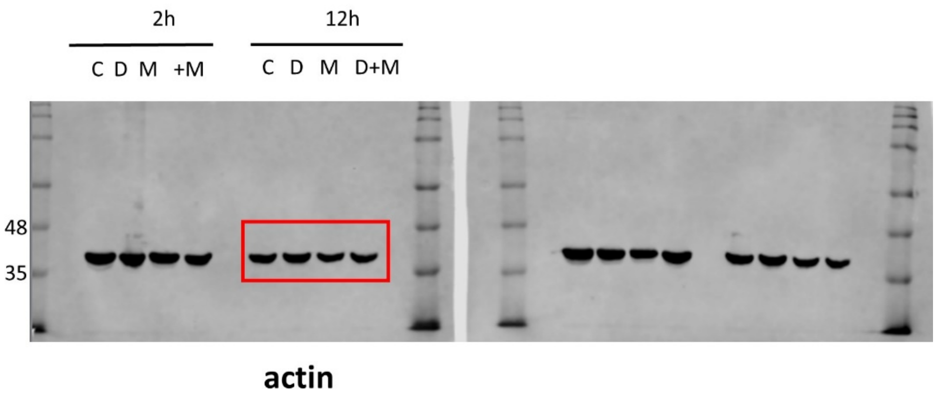

Figure S1. Western blot analysis of TWIST1, ER and actin. Uncropped membranes. Bands marked in the red rectangles were used for the composition of Figure 4.

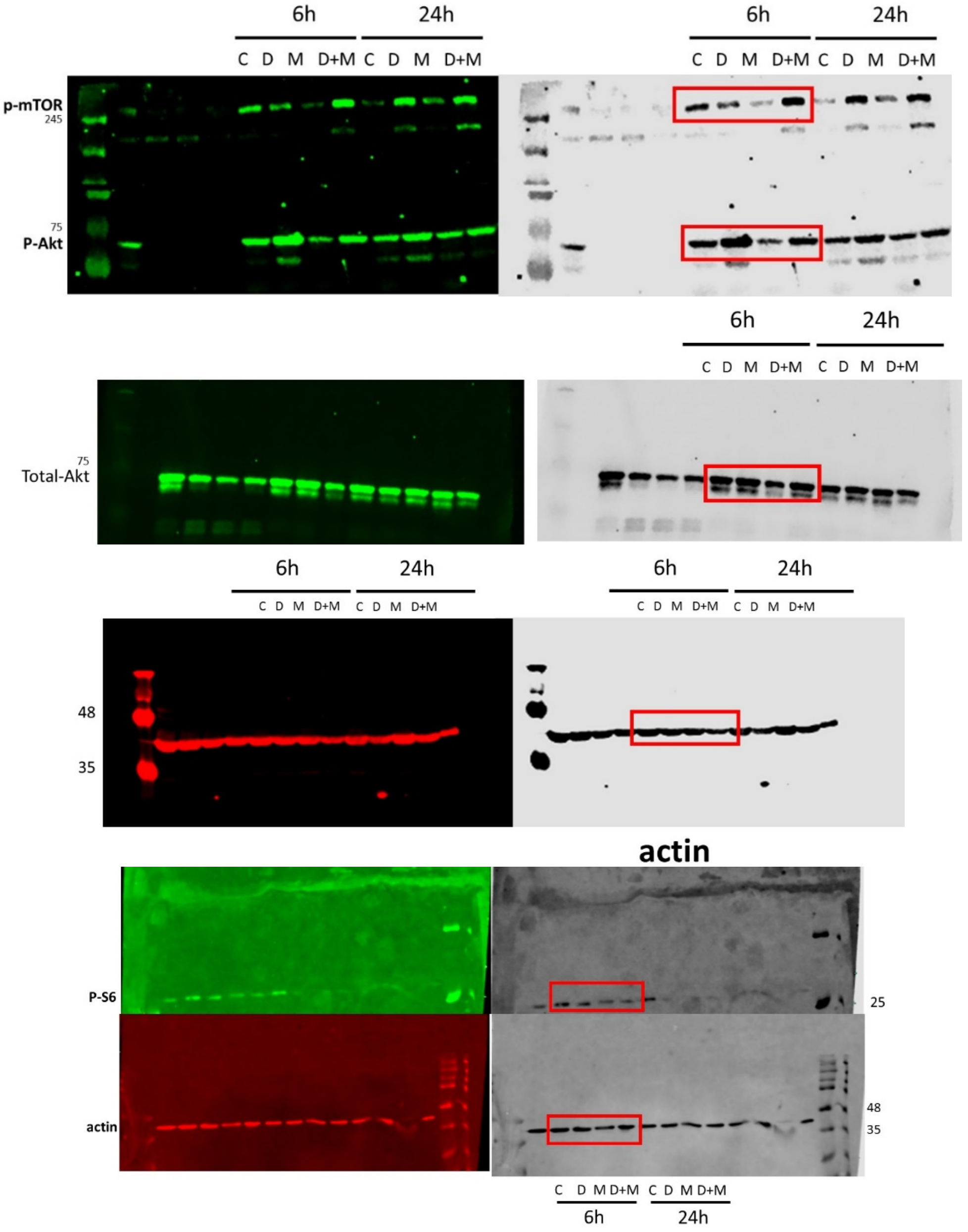

Figure S2. Western blot analysis of p-mTOR, Total Akt, p-Akt and actin. Uncropped membranes. Bands marked in the red rectangles were used for the composition of Figure 8.

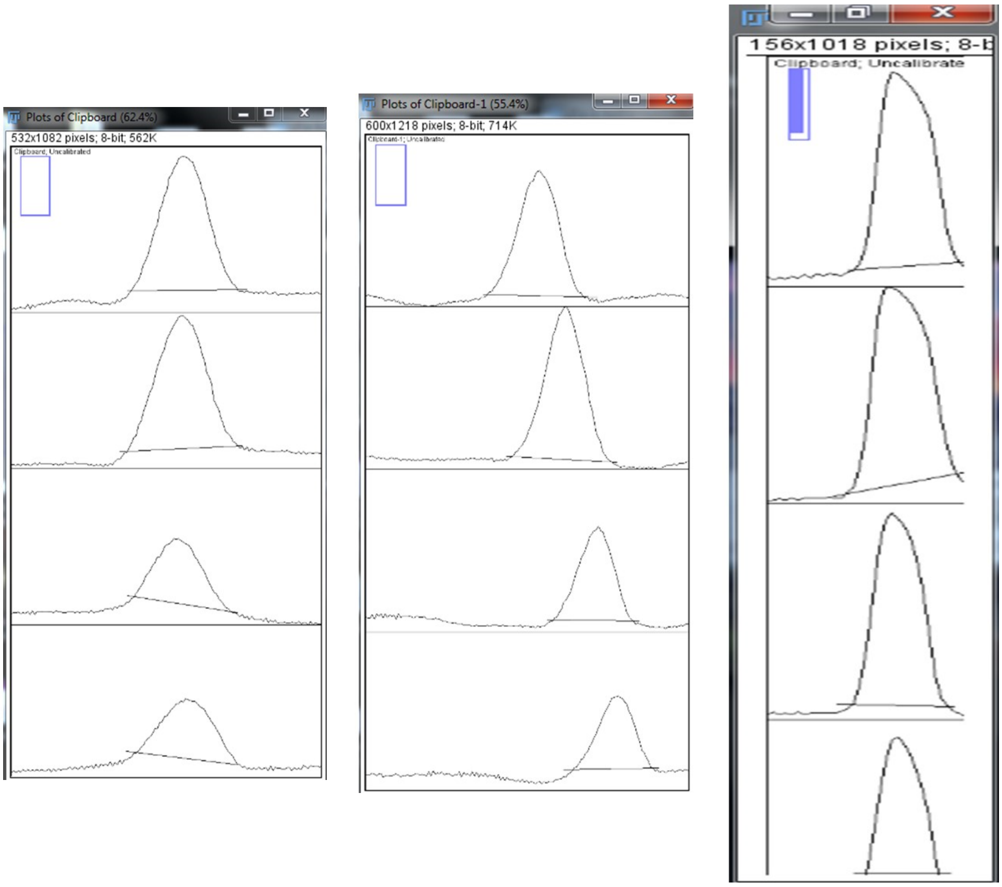

ER-alpha **Twist1** **actin**

Figure S3. Western Cuantification of the density of the bands included in Figure 4. It was made by using the image proccessing package ImageJ Fiji.

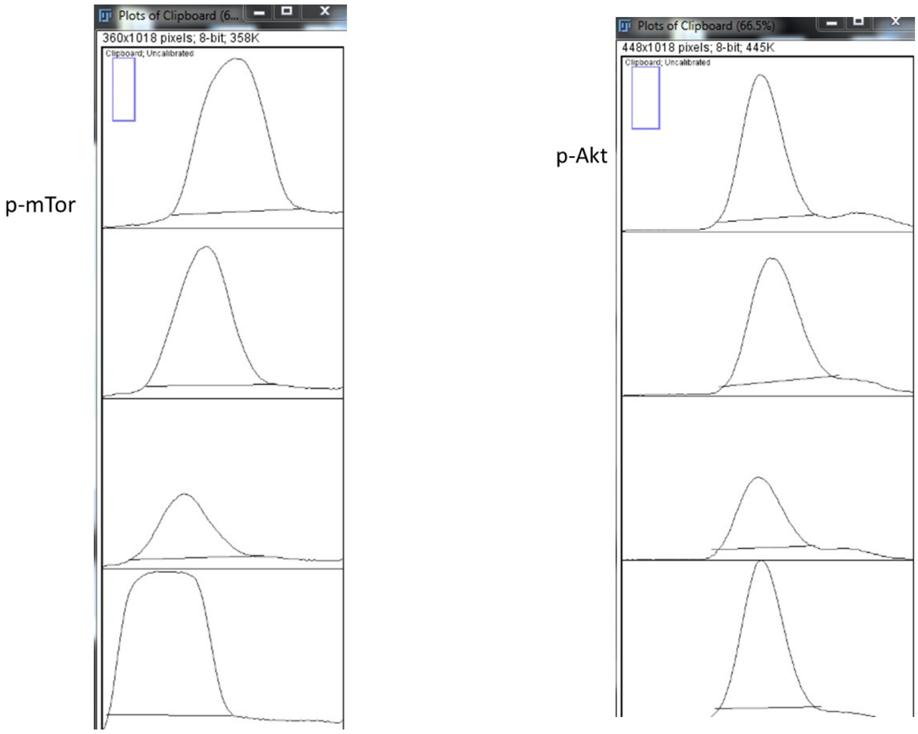

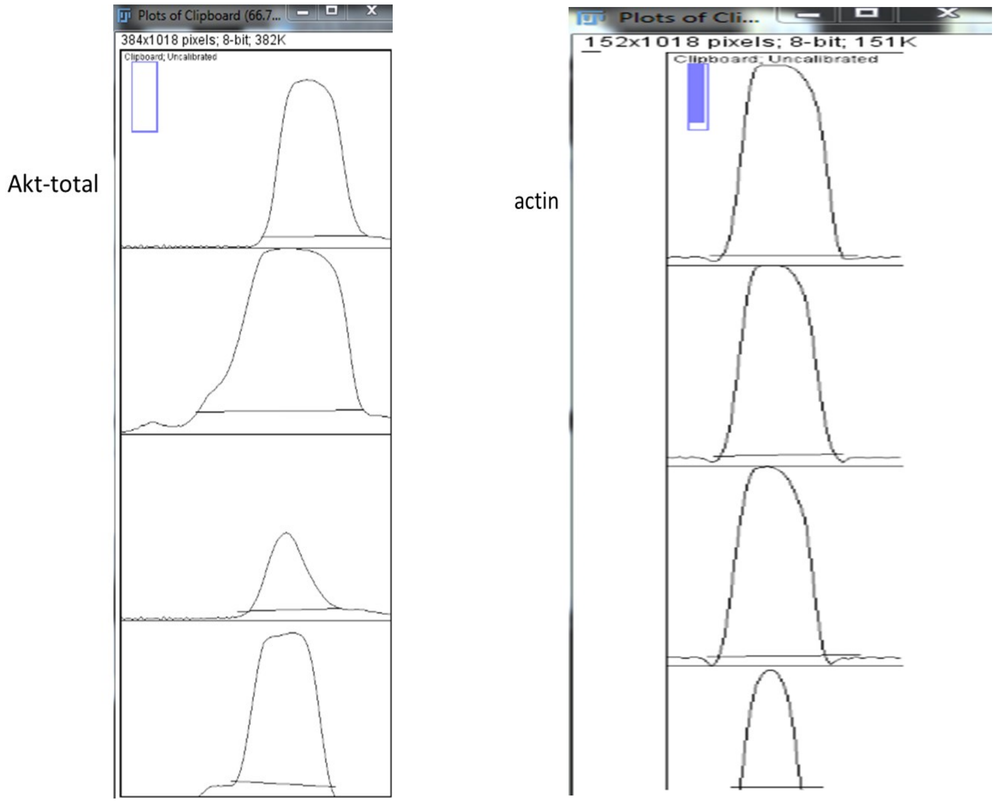

Figure S4. Western Cuantification of the density of the bands included in Figure 8C. It was made by using the image proccessing package ImageJ Fiji.

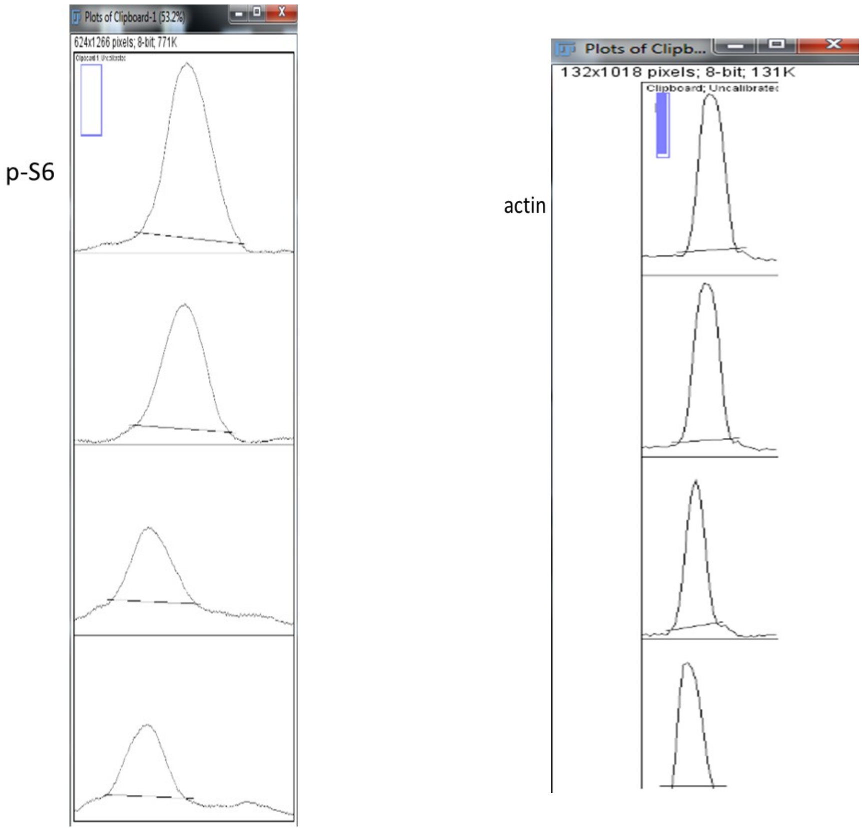

Figure S5. Western Cuantification of the density of the bands included in Figure 8D. It was made by using the image proccessing package ImageJ Fiji.

© 2019 by the authors. Licensee MDPI, Basel, Switzerland. This article is an open access article distributed under the terms and conditions of the Creative Commons Attribution (CC BY) license (http://creativecommons.org/licenses/by/4.0/).
